# The Appropriateness of Footwear in Diabetic Patients Observed during a Podiatric Examination: A Prospective Observational Study

**DOI:** 10.3390/jcm13082402

**Published:** 2024-04-20

**Authors:** Anisa Hazbiu, Ilaria Teobaldi, Mario Sepe, Giovanni Federici, Marco Meloni, Luigi Uccioli

**Affiliations:** 1 Ambulatorio CIMAU, Via G. Cesare 82, 66054 Vasto, Italy; anisanon@yahoo.it; 2 Independent Podiatrist, 37039 Verona, Italy; ilaria.teobaldi@gmail.com; 3 Centro Podologico Sepe, Via Alcide De Gasperi 4/D, 80036 Palma Campania, Italy; studio.sepe@libero.it; 4 Ospedale San Pietro Fatebenefratelli, Via Cassia 600, 00189 Roma, Italy; g.federici@hctdiabete.it; 5 Department of Systems Medicine, University of Rome “Tor Vergata”, 00133 Rome, Italy; luccioli@yahoo.it; 6 Division of Endocrinology and Diabetes, CTO Andrea Alesini Hospital, 00145 Rome, Italy

**Keywords:** adherence, TF, diabetic foot, podiatrist, prevention

## Abstract

**Background:** Adequate compliance with wearing therapeutic footwear (TF) to prevent diabetic foot ulcers is known to be low. The primary aim of this study was to identify population awareness about the ulceration and/or recurrence risk according to footwear choice. The secondary aim was to evaluate the compliance level in footwear choice based on a patient’s own risk. **Methods:** Forty podiatrists participated from 1 September 2017 to 31 August 2018, providing six-section forms which included personal data, risk classification, footwear characteristics and a knowledge questionnaire. **Results:** This study included 1507 patients. Those with active ulcers were excluded. A total of 43% of patients belonged to risk class 0, 19% to risk class 1, 19% to risk class 2 and 19% to risk class 3. A total of 58% had foot deformities. **Conclusions:** Nearly half of patients with a high risk of ulceration had knowledge of their own risk but the majority of them did not follow the recommendations. Only a small percentage (36%) of risk class 3 patients wore footwear suitable for their risk class. There was poor consideration of footwear choice among patients. We highlight critical issues in patient education and compliance with wearing footwear appropriate to their risk class.

## 1. Introduction 

The International Working Group on Diabetic Foot (IWGDF) calls attention to the prevention of diabetic foot ulcers (DFUs), which remains an ambitious goal to reach. Loss of protective sensation (LOPS), peripheral artery disease (PAD) and foot deformity are the major risk factors; additionally, a history of foot ulceration or any level of lower extremity amputation further increases the risk of ulceration up to 40% for one year after healing [1]. Pre-ulcerative lesions, quality of life, costs, foot-related mechanical stress and a patient’s compliance are also key factors in prevention of DFUs. People with diabetes without the above-mentioned risk factors and features do not appear to be at increased risk of foot ulceration compared to people without diabetes [2].

Investigators highlight, also, the relation between DFUs and depression. The emotional toll of limited mobility and the challenges of managing the condition can lead to frustration, hopelessness, and low self-esteem among individuals with DFUs. These negative emotions contribute to a poor compliance and increased preventable complications, such as amputations [3].

The five main pillars of foot ulcerative prevention have been indicated as follows [2]:

1. Identification of the at-risk foot.
2. Regular inspection and examination of the at-risk foot.
3. Patient, family and healthcare provider education.
4. Routine use of appropriate footwear.
5. Regular monitoring of risk factors for ulceration.

In addition, specific recommendations have been formulated in terms of footwear characteristics and use [2]:

1. Diabetic patients should always use accommodative, properly fitted therapeutic footwear (TF).
2. In presence of LOPS, the feet should be protected with the suggestion of not walking barefoot, not wearing footwear without socks and never using thin-soled slippers, both indoors and outdoors.
3. Patients with LOPS should have access to TF and should be encouraged to wear it all the day.
4. Patients with foot deformities should always wear TF accommodating their foot shape with appropriate fitting.

A prospective clinical trial revealed a significantly lower rate of DFUs in patients provided with therapeutic footwear compared to a control group [4]. Several studies reported that diabetic patients show adequate compliance with wearing TF and off-loading devices [3,4,5,6,7] in the presence of foot ulcers. Also, high re-ulceration rates are associated with non-compliance with the TF [8,9,10]. This is the reason that the first-choice treatments for foot off-loading in acute phases are non-removable devices [11,12].

There is no gold standard measure for TF compliance [13], but the objective methods such as the amount of daily steps, temperature sensors and activity monitors are the most usable and valid ways to obtain an estimate of TF compliance [5,14,15]. One of the major obstacles for compliance is the need to wear TF all day long [12,16], especially by patients in remission from a foot ulcer [17,18]. We can also cite several articles in the literature that demonstrate the importance of wearing TF whether indoors or outdoors [19,20].

According to the IWGDF Guidelines, TF is defined as footwear designed to have some therapeutic effect that cannot be provided by ordinary footwear. The characteristics of TF for an at-risk diabetic patient are that it be appropriately fitted, customized footwear, accommodative and demonstrated to have a plantar pressure relief effect during walking, and have extra depth, multiple width fittings and features designed to accommodate a broader range of foot types, modified out-soles, rocker bottom out-soles, fastenings and smooth internal linings, as well as inner space for custom-made insoles and in-shoe (semi)-rigid orthosis [2]. 

Therefore, any foot ulcer prevention measures cannot be successful without highlighting the central role of TF.

The purpose of this study was to answer two main questions:

(a) Is there enough patient knowledge on the basic principles of preventive footwear according to their foot ulcerative risk level?
(b) Do patients wear footwear adequate to their ulcerative risk level?

## 2. Materials and Methods

A total of 62 podiatrists, all members of the Diabetic Foot Study Group of the SID (Italian Diabetes Society), were involved in the recruitment of participants and data collection. The nearly 100 podiatrists registered in SID were contacted one by one via phone call, but only 62 decided to accept the invitation. All the podiatrists received formal training by the research supervising group (SG) on how to perform the data collection, and received an identification code “Centre ID”.

Eligible participants were naive people with diabetes (DPs), male and female, aged ≥ 18 years, attending a podiatric visit from 1st September 2017 to 31st August 2018. 

Verbal informed consent was obtained before starting any data collection. Based on the local policy, ethics committee approval was not required for the current observational study. This study was performed according to the Helsinki Declaration; patients’ data were anonymized, and privacy protected. 

A specific software was provided to all participants and was also published on the SID website at the following link: “https://www.siditalia.it/sid/gruppi/320-podopatia-diabetica#documenti-pubblicazioni”. Podiatrists unable to access the software were provided with a paper form. (Supplementary S1). All participants also received an explanatory legend on how to fill out the form and carry out the tests (Supplementary S2).

After the data collection, the trained podiatrists shared the data with the SG via email. 

The data collection comprised four stages:

1. Demographic data.
2. Definition of ulceration risk.
3. Types and characteristics of footwear (Supplementary S2 and S3).
4. Patient’s questionnaire.

### 2.1. Demographic Data

Sex, age, diabetes duration and education level were collected. In addition, their drug history and the clinician who referred the participants to the podiatrist were recorded.

### 2.2. Definition of Ulceration Risk

Participants were grouped according to their risk class as outlined in the IWGDF guidelines:

− Class 0 includes patients at very low risk of ulceration, without LOPS and without PAD.
− Class 1 includes patients at low risk of ulceration, with LOPS or PAD but without foot deformities.
− Class 2 includes patients at moderate risk of ulceration, with LOPS and PAD, or LOPS and foot deformity or PAD and deformity.
− Class 3 includes patients with LOPS or PAD and one or more of the following: histories of a foot ulcer, minor or major amputation.

PAD was defined by the absence of posterior tibial pulse and/or dorsalis pedis pulse. In the presence of peri-malleolar and/or dorsal edema that prevented palpation, the test was defined as not executable.

Neuropathy was defined by LOPS and evaluated by the monofilament test [21]. The test had to take place in a quiet place, with the monofilament applied perpendicular to the skin surface and with the patient not seeing when the filament was applied. Three points were tested on both feet: the big toe and the base of the 1st and of the 5th metatarsal. Protective sensation loss was detected if the participants were unable to feel two out of three points.

Foot deformities were considered by evaluating the foot shape mainly in the presence of overload, defined by the presence of significant callus on the plantar foot.

Any history of previous ulceration, Charcot neuro-osteoarthropathy and/or minor amputation was recorded.

### 2.3. Types and Characteristics of Footwear

The following shoes characteristics were recorded:

− Type a—Open: off-the-shelf sandals and similar (flip-flops, sandals, open slippers).
− Type b—Closed: off-the-shelf standard closed shoes (moccasins, décolleté, slippers, boots).
− Type c—Sneakers: off-the-shelf sporty and gymnastic footwear.
− Type d—Therapeutic footwear: off-the-shelf or custom-made, designed to accommodate customized foot orthosis, without internal seams, with rigid or semi-rigid out-sole and flexible, elastic or self-modeling upper, extra depth and rocker bottom out-sole.

Out-sole—Flexible, biomechanical semi-rigid and rigid sole.

Upper—Thermoformable, elastic, self-modeling and rigid.

Wrong size or fit—The footwear size was checked by inserting the finger between the back of the foot and the shoe, evaluating both too long and too short sizes. The fit was checked with a tape measure, measuring the shoe and the metatarsal diameter.

Internal seams—The presence of internal seams was checked by inserting the hand inside of the footwear, checking the entire internal surface, paying more attention to the forefoot.

Predisposed to insole—Footwear designed to accommodate insoles.

Insole—The presence of orthotic devices was evaluated, and these were classified as customized, preformed and off-the-shelf insoles.

After the footwear assessment, the trained podiatrists evaluated whether the footwear was appropriate for the patient’s risk of foot ulceration based on the IGWDF recommendations. In detail, the *Guidelines* recommend prescribing for patients as follows:

− For IWGDF risk 1–3 with no or limited foot deformity, no pre-ulcerative lesions and no plantar ulcer history, shoes that accommodates the shape of the feet and that fit properly.
− For IWGDF risk 2 or 3 with a foot deformity that significantly increases pressure or a pre-ulcerative lesion, extra-depth shoes, custom-made footwear, custom-made insoles and/or toe orthoses.
− For IWGDF risk 3 with a healed plantar foot ulcer, therapeutic shoes that have a demonstrated plantar pressure relieving effect during walking, to help prevent a re-current plantar foot ulcer.

### 2.4. Patient’s Questionnaire

A 10-question questionnaire, with nine closed questions and one open question, as shown in Table 1, was provided to each participant. The questions dealt with their knowledge and awareness about the basic principles of prevention and the role of footwear and were divided as follows:

− Four questions related to education on footwear.
− Four questions related to patient awareness about the role of footwear.
− Two questions on adherence.

**Table 1:** Footwear questionnaire.

1	The footwear you are wearing today was recommended by someone? □ YES □ NO From who? □ Endocrinologist □ Podiatrist □ Physiatrist □ Orthopedic technician □ Orthopedic □ Other: _____________________
2	Do you think you are wearing suitable footwear? □ YES □ NO
3	How many hours a day do you wear this footwear? ________________
4	Do you think foot ulcers can come from footwear? □ YES □ NO
5	Have you ever been told your feet are at risk of (re)ulceration? □ YES □ NO
6	Do you think they are? □ YES □ NO
7	Have you ever received any recommendation on choosing footwear? □ YES □ NO
8	Do you remember at least three? 1______________________ 2 _______________________ 3 ______________________
9	Can you follow these recommendations? □ YES □ NO
10	Do you think they are excessive? □ YES □ NO

As far as known by the authors, no validated questionnaire for footwear compliance in patients with diabetic foot and at risk of foot ulceration is currently available in the literature. The questionnaire used in this study was designed by a team of clinicians with extensive experience in diabetic foot care, in order to produce an easy-to-use form in a clinical setting and specifically designed to address the aims of the current study. This questionnaire has not been validated and this is one of the limitations of this study.

### 2.5. Statistical Analyses

Statistical analysis was performed by using SAS (JMP12; SAS Institute, Madison, WI, USA).

Descriptive statistics was used to analyze the demographic data. Continuous variables were expressed as mean and standard deviation, while categorical variables were presented as percentage or proportion.

A chi-squared test was used to highlight differences among different risk class groups. *p*-values < 0.05 were considered significant.

## 3. Results

Only 40 trained podiatric centers sent data during the study period. Of these, 61% were public hospitals, 29% private structures and in 10% of the cases the data were collected in both public and private structures.

More than 1800 patients were observed but several datasheets were rejected because poorly compiled; for this reason, only 1766 datasheets were collected.

### 3.1. Demographic Data

Patients had an average age of 71 ± 11.5 years, mainly male (56%), with an average duration of the diabetes of 17 ± 11 years. A total of 52% had a low education level, and 48% of the patients were insulin-treated and had more years of disease (*p* < 0.0001) (Table 2).

A total of 60% of patients were sent by endocrinologists. A total of 24% of patients had a previous ulcer and/or minor amputation and 2% had previous Charcot neuro-osteoarthropathy correlated by written documentation or self-reported by patients. A total of 15% had an active ulceration at the observation time and were excluded from the study; for this reason, the rest of the statistical evaluation was carried out on 1507 datasheets. A total of 58% had a foot deformity like claw/hammer toes, hallux valgus or cavus or flat foot. The presence of deformity was significatively higher in risk class 3 patients (73%) compared to other risk classes (*p* < 0.0001).

### 3.2. Ulceration Risk Class

**Figure 1:**
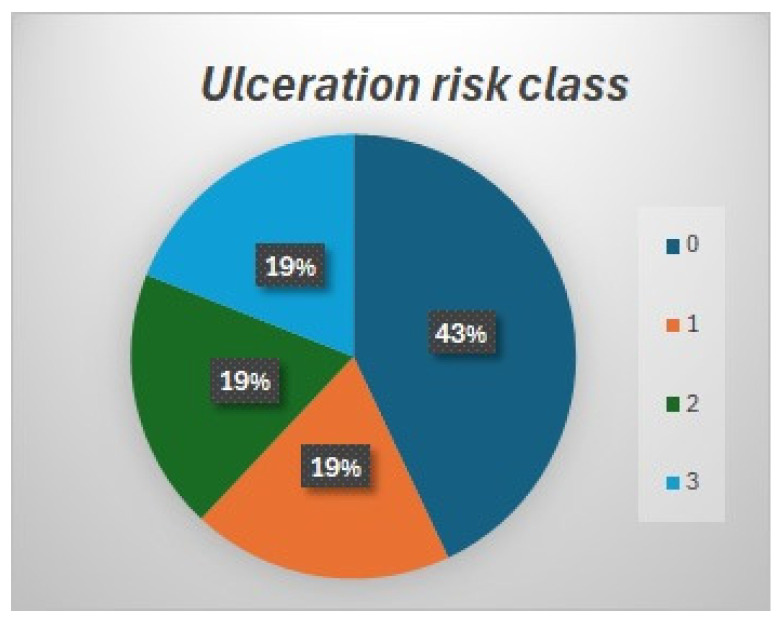
Patient distribution by ulceration risk class.

According to the IWGDF risk classification, patients were divided as follows: 43% in class 0, 19% in class 1, 19% in class 2 and 19% in class 3. As expected, the patients in class 0 were more than those belonging to classes 1, 2 and 3, which had a homogeneous distribution (Figure 1).

### 3.3. Patient Questionnaire

**Figure 2:**
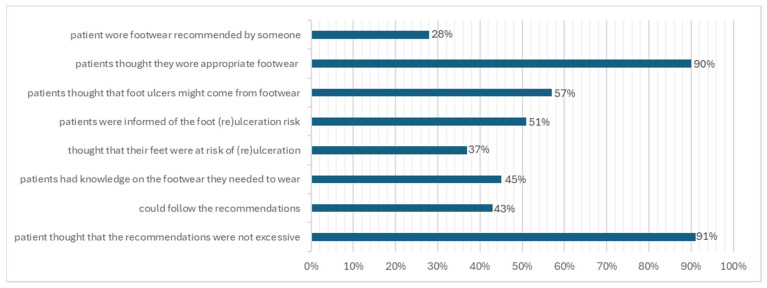
Questionnaire answers.

Patients answered the questionnaire as follows (Figure 2):

1. Only 28% of patients wore footwear recommended by someone, principally by endocrinologists (13%) and podiatrists (9%)
2. A total of 90% of the patients thought they wore appropriate footwear.
3. Patients wore the footwear for an average of 8 ± 3.9 h a day.
4. A total of 57% of patients thought that foot ulcers might come from footwear.
5. A total of 51% of the patients were informed about foot (re)ulceration risk.
6. A total of 37% thought that their feet were at risk of (re)ulceration.
7. A total of 45% of patients had knowledge of the footwear they needed to wear.
8. Mostly, the recommendations were to wear TF, with a flexible upper, without internal seams, predisposed to insole, etc.
9. A total of 43% could follow the recommendations.
10. A total of 91% of patients thought that the recommendations were not excessive.

Definitely, 37% of all patients knew that they were at risk of ulceration, and among them, the majority of patients wearing TF (62%) belonged to risk class 3.

### 3.4. Types and Characteristics of the Footwear

**Figure 3:**
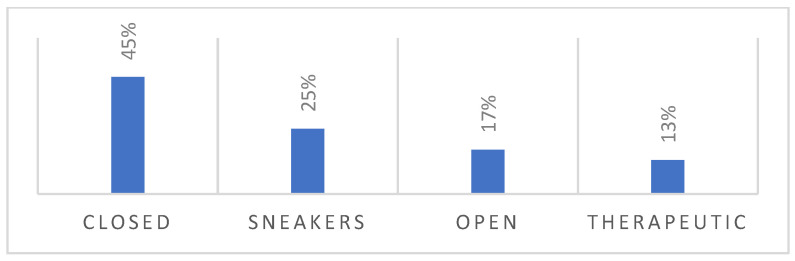
Percentages of observed footwear.

**Figure 4:**
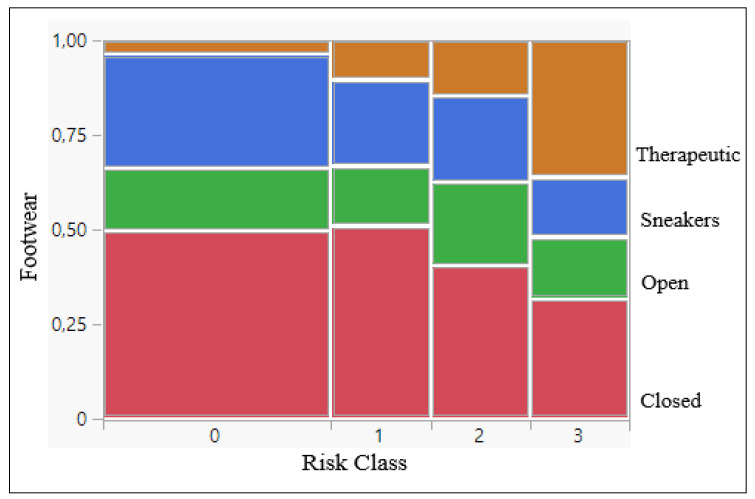
Footwear contingency analysis by risk classes (*p* < 0.0001).

According to the survey, 45% of the patients observed wore off-the-shelf closed footwear, 25% off-the-shelf sneakers and 17% off-the-shelf open footwear. Only 13% wore TF (Figure 3 and Figure 4).

From the data in Table 3, we observed that in risk class 3 the presence of TF was significantly higher compared to the other risk classes (*p* < 0.0001).

Out-Sole—Observing the type of out-sole by risk class, we noted that in risk class 3 there was a significantly higher percentage (35%) of rigid out-soles compared to in other risk classes, where we found more flexible out-soles (*p* < 0.0001).

Upper—In risk class 3, there was a significantly greater presence of thermoformable/elastic and self-modeling uppers and a lower percentage of rigid uppers compared to the other risk classes (*p* < 0.0001).

Wrong size or fit—A total of 12% of patients wore footwear with the wrong size or fit (7% in risk class 3).

High-heel footwear—A total of 6% of the patients wore high-heel footwear (>4 cm) (5% in risk class 3).

Internal seams—A total of 36% of the patients wore footwear with internal seams (17% in risk class 3).

Predisposed to insole—A total of 44% of the footwear observed was predisposed to foot orthoses. We found that 29% of the footwear was not predisposed to foot orthoses in risk class 3.

## 4. Discussion

The results of our study showed that more than half of patients—57%—were aware of the correlation between their foot ulcers and inappropriate footwear and 51% of them had been instructed on foot (re)ulceration risk. This level of awareness about the regular use of TF is fundamental because, as explained by Alkhatieb MT. et al. [22] in a recent cross-sectional study, the DFU recurrence rate among patients who use TF was lower than in patients who did not (27.8% vs. 52.5%).

In addition, 90% of patients thought they wore appropriate footwear according to their ulceration risk, but among them, we found that only 36% of patients in risk class 3 wore TF. This last result highlighted the patients’ misperceptions of the footwear which they wore. Again, from our study, more than half of patients had no knowledge of the footwear they needed to wear, and this is an unsatisfactory percentage considering that 25% of them had a medium–high risk of ulceration. These numbers support the idea that they did not receive adequate information about their foot condition. Probably, this result is due to the patients’ attention to foot care and self-management, or the issue of foot care for healthcare professionals may be more important than TF. However, patients with diabetes often do not receive close attention to foot care by their healthcare providers [23]. A combination of qualified healthcare personnel and patient education, TF, prevention and regular monitoring can lower amputation rates by 49–85% [24].

The perception of the foot problem was a focal point of our study. While TF is not effective to enhance healing, it is mandatory for the prevention of first ulceration and recurrences [25]. Overall, 37% of all patients thought that their feet were at risk of (re) ulceration, and in risk class 3, 62% of patients were found to use TF. These data seem to show that the patients at highest risk are more aware of the risk of foot (re)ulceration and more compliant to therapeutic recommendations. Alkhatieb MT. [22] found that 47% of patients with high ulceration risk wear TF and 46% were compliant with wearing it. Knowles and Boulton [6] recommended that diabetic patients need to understand their foot condition severity and the benefit of wearing TF. Based on our study, almost half of patients were advised on the appropriate footwear they needed to wear, but 57% were not able to follow the recommendation despite almost everyone believing that the advice was not excessive.

Compliance with wearing TF is positively influenced only if two prerequisites are fulfilled: the awareness that the foot condition is a problem, and a perceived benefit and acceptance of wearing the appropriate footwear. TF has been described as a “visible representation of the disease”, and often addresses the patient’s underlying diabetic foot problem [26]. For this reason, and also because López-Moral M. et al. [27] in a prospective multicenter study found that the adherence to TF results in fewer ulcerations, patient information and education about it is crucial [25].

From our data, patients in risk class 3 wore TF for about 8.6 h a day, nearly 70% of the daytime. Jar G. et al. [5] found a median wearing time of the TF of 8–12 h a day. High-quality evidence shows that consistent use of TF prevents the recurrence of plantar ulcers, specifically of the metatarsal heads [17,28], which can reduce plantar peak pressure by approximately 50% when worn more than 60% of the day [29].

It is the combination of adequate pressure relief and compliance with wearing the footwear that gives the best clinical effectiveness [28], but the effects of various preventive interventions are reduced by low adherence to the recommended treatment [30]. This is in line with our results which show that 70% of the patients in risk class 3 wore footwear recommended by a specialist but only 36% of them wore suitable footwear. Recently, Sudha B. G [13], in a cross-sectional study, collected information on foot care education retention using a 10-item questionnaire similar to the one used in our study. Only 30.5% of patients acknowledged receiving foot care instruction, and their foot care education retention rate was high for only 12%. That author listed barriers for good foot care. In particular, the lack of awareness in diabetic patients is influenced by a lack of financial assistance, the travel time and expenses involved in reaching a foot clinic, family dependencies, religious practices promoting walking barefoot and a communication gap with healthcare professionals.

People with diabetes should wear footwear that fits, protects and accommodates the shape of their feet. This includes adequate length, width and depth, and consequently adequate girth and volume [31]. Many patients with diabetes wear footwear that does not fit appropriately [32]. Only 17% of our patients in risk class 3 wore footwear with internal seams, and 7% wore footwear with the wrong size or fit. Also, our patients in risk class 3 showed a greater presence of flexible/elastic/self-modeling uppers and a significantly higher percentage of footwear with rigid out-soles, compared to other risk classes. López-Moral M. el al. [27] reported that patients with high risk of ulceration who used a rigid rocked sole had a 64% lower risk of developing a recurrence compared with patients who used a semirigid sole. This result confirms that inappropriate footwear is a common trigger for foot ulceration, as it exposes patients to the direct effects of pression, shear-stress friction and/or irritation [19].

TF is more effective than conventional footwear in reducing peak pressure in the forefoot area in diabetic patients [33]. From our observational data, only 36% of patients in risk class 3 wore TF. Uccioli et al. [34] demonstrated a significant reduction in the incidence of foot ulcers in patients with high risk of ulceration that were treated with manufactured therapeutic shoes and insoles compared to a second group that used self-selected shoes (58.3 % vs. 27.7%). It is important to highlight that foot orthotics are as important as the shoes in which they are worn [35]. In our study, we looked at the type of footwear and orthotics worn by patients in risk class 3, and we noticed that the patients who wear TF almost all also wear customized orthotics. This observation shows that prescriptions for TF in patients with a high risk of ulceration are accompanied by customized orthotics.

In order to increase compliance, some authors proposed recently an “intelligent footwear design” that resembles conventional footwear. They also suggested the use of a removable pressure-sensing system that detects the location of high plantar pressure and correspondingly adjusts the contour of the insole. This technological innovation still needs to be evaluated through clinical trials that will confirm its effectiveness [22].

Furthermore, adhering to clinical practice guidelines, routine assessment, screening and treatment of depression in patients with diabetes is recommended [10,36]. Patients suffering from depression are less likely to follow ulcer care; however, artificial intelligence (AI) monitoring including smart socks and insoles, which can reduce the risk of ulcer formation and improve patient adherence, have facilitated patients maintaining adherence to care [3]. Also, it is important to highlight emerging technologies as potential facilitators in patient compliance, such as infrared thermography, plantar pressure, as well as mobile foot care facilities, remote monitoring systems and rehabilitation centers for patients living in rural areas [13].

The awareness around the benefit of wearing appropriate footwear, the importance of aesthetics, and personal perception, values and experiences are important factors which appear to influence compliance [25]. We all need to be prepared to amend, repeat and reinforce messages in order to support patients over a lifetime of behavioral change [37].

There is scant research on interventions to improve TF compliance [28], Keukenkamp R. et al. [38] showed that motivational interventions (MIs) also have short-term positive effects. Podiatrists work directly on diabetic foot care and with high-risk diabetic patients and can motivate them to better self-care on a regular basis. There is a great opportunity for podiatrists to explore motivational interventions to increase acceptance and compliance with regard to wearing appropriate footwear [39]. In addition, the podiatrist can be the central figure of this educational process for the diabetic patient, and should implement effective educational programs in short- and long-term periods.

## 5. Conclusions

There is a lack of information and awareness on the need and effectiveness of wearing therapeutic footwear to prevent foot ulceration. Even in the case of specific indications, it appears that patients do not follow clinicians’ recommendations.

Just over a third of patients in the high-risk class wear footwear suitable for their foot risk. There is poor compliance with prescribed footwear and low awareness of its usefulness.

We would suggest that healthcare professionals reinforce education and adjust the behavior of non-compliant patients by promoting educational programs and constantly re-evaluating their compliance with the prescribed footwear.

## Figures and Tables

**Table 2:** Patient baseline characteristics.

Age (average ± SD)	71 ± 11.5
DM duration (years ± SD)	17 ± 11
Sex (% male)	56
Insulin-treated (% yes)	48
Education level (% low)	52
Charcot (% yes)	2
Previous ulcer/amputation (% yes)	24
Active ulceration (% yes)	15
Deformity (% yes)	58

(Abbreviations: SD: standard deviation; DM: diabetes mellitus).

**Table 3:** Percentages of footwear by risk class.

Risk Class	Closed	Open	Sneakers	Therapeutic
0	45.5%	17%	30%	3.5%
1	51%	16%	23%	10%
2	41%	22%	22.5%	14.5%
3	32%	16%	16%	36%

## Data Availability

Data were recorded by authors in a local database. Data are not available because of privacy and ethical restrictions.

## Supplementary Materials

The following supporting information can be downloaded at: https://www.mdpi.com/article/10.3390/jcm13082402/s1, Supplementary S1. Data Collection Sheet. Supplementary S2. Legend. Supplementary S3. Footwear Legend.
