# Supplementary material for: The Appropriateness of Footwear in Diabetic Patients Observed during a Podiatric Examination: A Prospective Observational Study
Source: J Clin Med. 2024 Apr 20;13(8):2402. doi: 10.3390/jcm13082402 (PMC11051551; doi:10.3390/jcm13082402)

Supplementary Materials

Supplementary S1. Data Collection Sheet

DATA COLLECTION SHEET FOOTWEAR OBSERVATIONAL STUDY

DATE ___________________
CENTER ID ____ DATE OF BIRTH ____________ PATIENT INITIALS______
SEX □ M □ F **YEARS OF DIABETES ____ INSULIN THERAPY** □ YES □ NO
EDUCATION LEVEL: □ LOW □ MIDDLE □ HIGH
SENT BY: □ ENDOCRINOLOGIST/DIABETOLOGIST
□ GENERAL MEDICAL DOCTOR □ OTHER
MONOFILAMENT TEST: RIGHT □ PRESENT □ABSENT
LEFT □ PRESENT □ABSENT
PULSE PALPATION TEST: RIGHT □ PRESENT □ABSENT
PEDIDIUM LEFT □ PRESENT □ABSENT
PULSE PALPATION TEST: RIGHT □ PRESENT □ABSENT
POSETERIOR TIBIAL LEFT □ PRESENT □ABSENT
DEFORMITY: □ PRESENT □ABSENT
PREVIOUS ULCERATION/AMPUTATION: □ RIGHT □ LEFT
IF YES, SPECIFY THE LOCATION ___________________________
ACTIVE ULCERATION: □ RIGHT □ LEFT
PREVIOUS CHARCOT: □ RIGHT □ LEFT
RISK CLASS: □ 0 □ 1 □ 2 □ 3

FOOTWEAR WORN AT THE TIME OF VISIT
Open Slippers □ Sandals □ Flip-flops □
Closed Décolleté □ Moccasins □ Classic □ Sneakers □
Boots/Ankle boots □
Gymnastic □
Therapeutic □
Injury brace □
Other □
OTHER CHARACTERISTICS
DESINGNED FOR INSOLES □
HEEL > 4 CM □
WRONG SIZE AND/OR FIT □
INTERNAL SEAMS □
PRESENCE OF SOCKS □

FLEXIBLE SOLE □
SEMI-RIGID BIOMECHANICAL SOLE □
RIGID BIOMECANICAL SOLE □
ROCKING SOLE □
TERMOFORMABLE/SELF-MODELING UPPER □
ELASTIC UPPER □
RIGID UPPER □

OTHER □
FOOT ORTHOSIS
ORTHOSIS CLASSIFICATION: □ Custom-made □ Preformed □ Insoles
RECOMMENDED BY: □ Endocrinologist/Diabetologist □ Podiatrist
□ Physiatrist □ Orthopedic Technician
□ Orthopedist □ Other: __________________

POOR HYGIENE
External footwear □
Internal footwear □
Socks □
Feet □

QUESTIONNAIRE

(See Table 1 main test)

Supplementary S2. Legend

LEGEND FOR THE FORM COMPILATION OF FOOTWEAR OBSERVATIONAL PROTOCOL

Education Level:
Low: primary and secondary schools (elementary and middle schools);
Middle: secondary schools (high schools);
High: Bachelor's degree.
Pulse palpation test:
Palpate the pedidium pulse and posterior tibial bilaterally,
N.E. (not executable) if there is peri-malleolar and/or dorsal edema that prevents palpation
The item "PRESENCE OF ULCER" should be left blank if ABSENT.
Monofilament test:

- The test should take place in a quiet, relaxed place.
- First apply the monofilament to the patient's hands (or elbow or forehead), so that the patient knows what to expect.
- The patient must not see if the filament is applied.
- There are at least 3 points to test on both feet: big toe, base of the 1st and 5th metatarsals.
- Apply the monofilament perpendicular to the skin surface.
- Apply enough force for the monofilament to flex.
- The total duration of the test, from the moment the monofilament comes into contact with the skin to when you remove it, should be approximately two seconds.
- In the presence of an ulcer, callus or scar, DO NOT apply the filament above, but near the interested area.

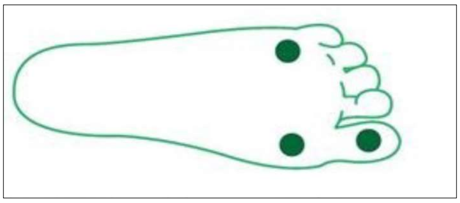

- Do not slip the filament on the skin and avoid repeated contact with the area on which the test is carried out.
- Press the filament against the skin and ask the patient if they feel pressure (yes/no) and then where they feel it.
- Repeat the procedure twice at the same point, and alternate it with one simulation, during which the filament is not applied (in total three questions per point).
- Protective sensation is present at each point if the patient responds correctly in two out of three applications.
- The protective sensation is absent with two out of three incorrect answers and the patient must therefore be considered at risk of ulceration.
- Encourage the patient during the test.

Suspected Risk Class:
0: no complications;
1: neuropathy;
2: neuropathy + vasculopathy and/or deformity;
3: neuropathy + vasculopathy and/or deformity, previous ulceration/amputation and/or previous Charcot.

Footwear

FOOTWEAR WORN AT THE TIME OF VISIT	
OPEN (See photos)	Slippers: off-the-shelf, WITHOUT back buckle, open or closed on the toes. Examples: rubber swimming slippers, with double and crossed buckles, Crocs, cloth house slippers, leather slippers, terry slippers. * Exceptions include: cloth slipper with zipper, closed on the toes and back. Sandals: off-the-shelf, WITH back buckle, open or closed on the toes. Examples: Birkenstock, in leather or rubber or other material, elegant or sporty. Flip-flops: off-the-shelf, Havaianas and similar, sporty and elegant.
CLOSED (See photos)	Décolleté: off-the-shelf, closed or open toe and back. Examples: ballerina footwear, elegant pointed shoe. Moccasins: off-the-shelf, classic for men or women, low uppers on toes, with rubber sole or other materials. Example: boat footwear. Classic: off-the-shelf, classic men's or women's shoe in leather or other material with a low fit, more or less elegant. Example: Parisian footwear. Sneakers: off-the-shelf, any model not included in the other categories, casual, more or less elegant or sporty footwear but not suitable for gymnastic activities. Examples: Camper, Converse All Star, low at the ankle. Boots/Ankle boots: off-the-shelf, with high ankle length. Examples: Timberlands, ankle boots, rain boots, Converse All Star high ankle.
GYMNASTIC	Off-the-shelf models for SPORTS activities: running, jogging, trekking.
THERAPEUTIC	Preventive suitable footwear, custom-made footwear built by orthopedic technician, specialized.
INJURY BRACE	Temporary footwear/braces for active injuries and bandages. Examples: Optima, Teradiab, Talus, Teraheel.
OTHER	Report only in case of real impossibility of assigning the footwear to previous classes.

Other Characteristics	
Custom-made insole	This category includes preventive/TF and any open/closed shoe with a removable and replaceable custom-made insole.
Wrong size and/or fit	Report both too long and too short sizes (put the finger between the back of foot and the footwear, evaluating the space) and wrong fit (with a tape measure, the fit of the shoe and the metatarsal diameter).
Heels > 4 cm	Report only if higher than 4 cm.
Flexible sole	Normal sole, totally flexible at the level of the metatarsals.
Biomechanical sole semi-rigid	Sole that resists flexion partially during the propulsion phase of the step and bends slightly.
Rigid biomechanical sole	Sole that totally resists bending during the propulsion phase and does not bend at all.
Rocking sole	Sole with early rolling point, i.e., before the metatarsal heads.
Rigid upper	Rigid materials.
Internal seams	Not to be confused with the external ones: put your hands inside the footwear and personally check.
Presence of socks: Type	Valid: cotton, wool, sponge, microfiber, plant materials. Invalid: nylon.
Other: Report any characteristic that does not belong to the previous ones and which may be risk of ulceration.	

Foot orthoses:

<u>Custom-made:</u> thermoformable foot orthoses, or plaster cast from phenolic foam or plaster bandage, leavened, cad-cam and similar.

<u>Preformed:</u> semi-finished, already assembled, ready
(e.g., gel insoles, carbon insoles, standard insoles present inside the footwear).

<u>Insoles:</u> insoles purchased by the patient himself in healthcare shop, pharmacies or elsewhere.
<u>Recommended by:</u> recommended and/or prescribed through the SSN (National Health System).
Poor hygiene:
Report any presence of dirt, dust, hair of animals, hair, blood stains or other things present inside and outside the shoe, with the exception of the outer sole.
Questionnaire:
Ask the questions exactly as they are shown on the questionnaire so as not to influence the patient's response.
<u>Question 9:</u> Briefly note which advice patients remembers, without suggestions or indications and only those relating to footwear, not to foot.

Supplementary S3. Footwear Legend

Sample of images
SLIPPERS

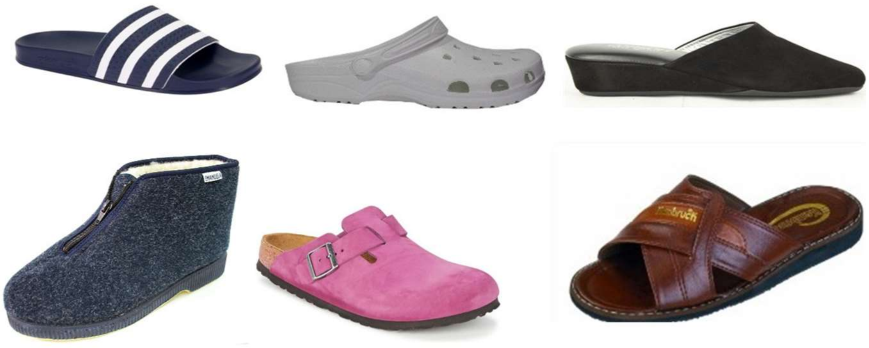

SANDALS

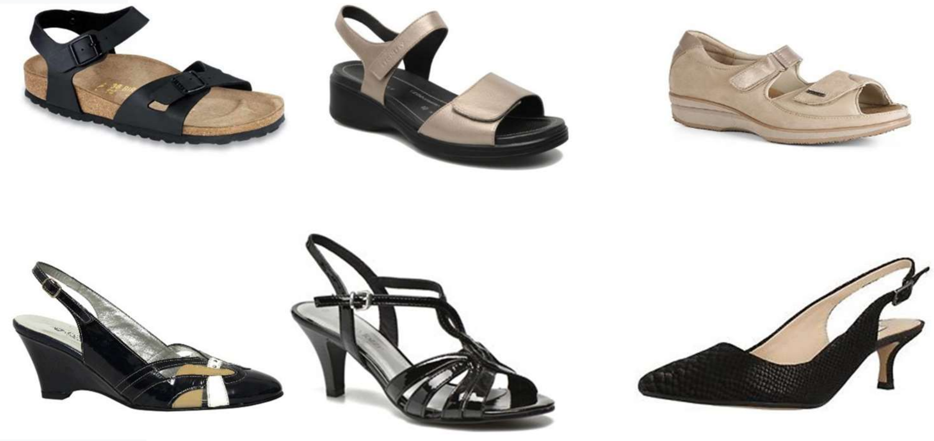

FLIP-FLOPS

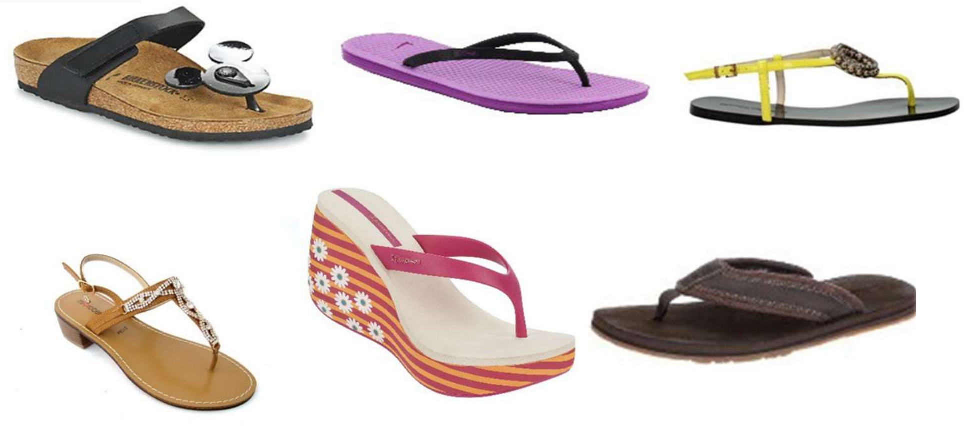

DE'COLLETE'

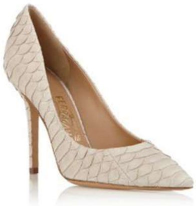 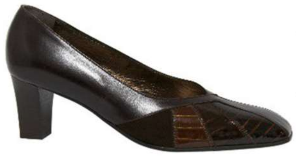 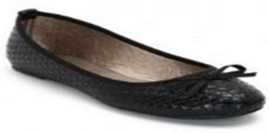

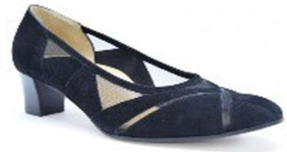 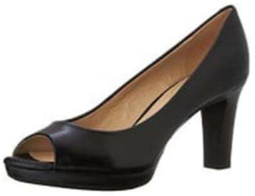

MOCCASINS

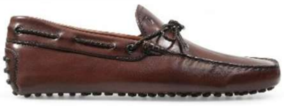 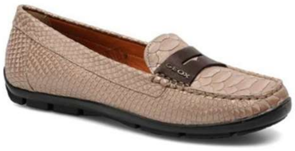 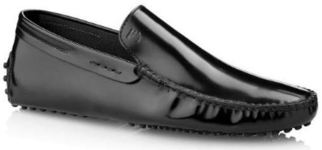

CLASSIC FOOTWEAR

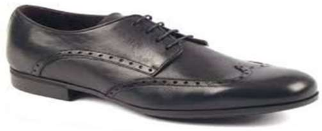 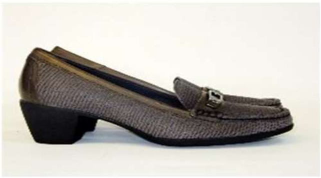 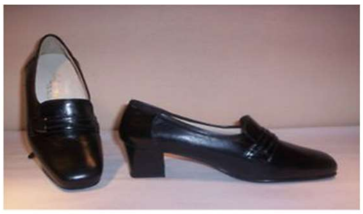

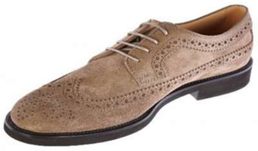 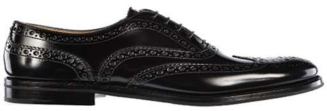 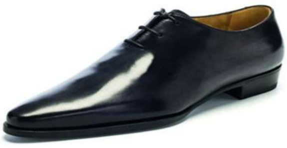

SNEAKERS

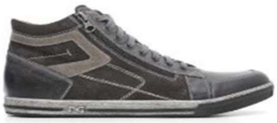 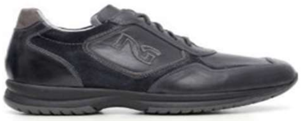 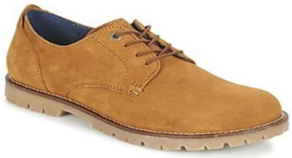

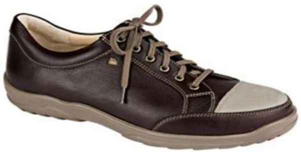 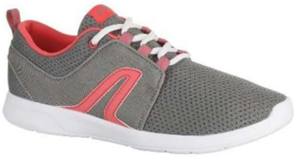 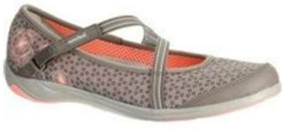

BOOTS

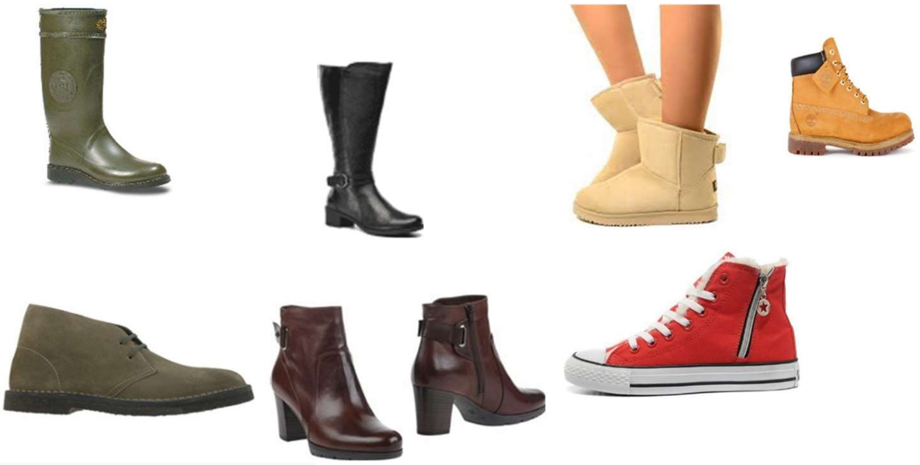

GYMNASTIC FOOTWEAR

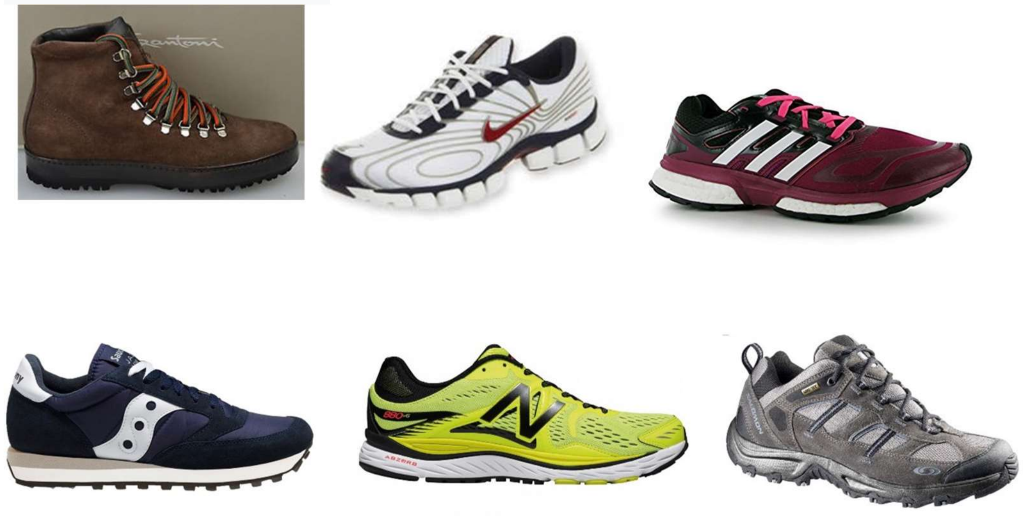

INJURY BRACE

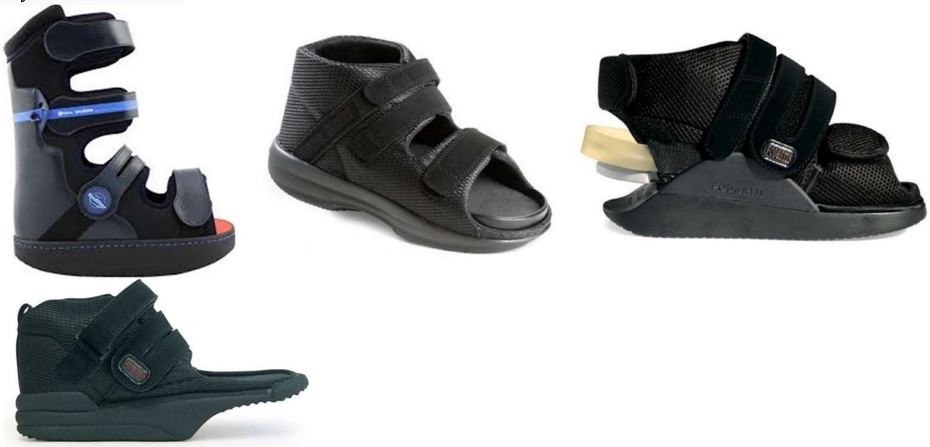

THERAPEUTIC FOOTWEAR

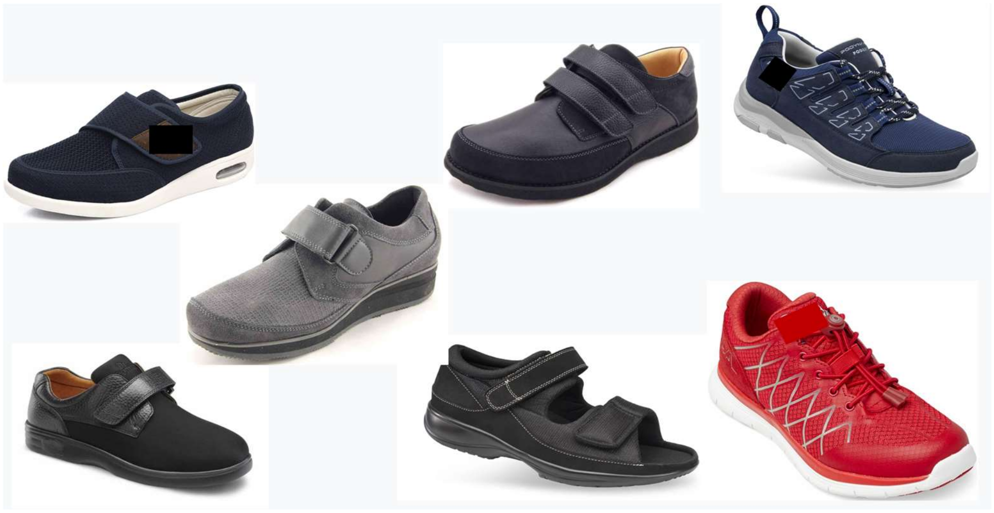

Supplement: Supplementary file 1 [file jcm-13-02402-s001.zip › jcm-2905618-supplementary.pdf]
